# Registration of randomized controlled trials in nursing journals

**DOI:** 10.1186/s41073-017-0036-9

**Published:** 2017-07-16

**Authors:** Richard Gray, Ashish Badnapurkar, Eman Hassanein, Donna Thomas, Laileah Barguir, Charley Baker, Martin Jones, Daniel Bressington, Ellie Brown, Annie Topping

**Affiliations:** 1 School of Nursing and Midwifery, La Trobe University, Melbourne, Australia; 2 Academic Health System, Hamad Medical Corporation, Doha, Qatar; 3 School of Health Science, University of Nottingham, Nottingham, UK; 4 Department of Rural Health, The University of South Australia, Adelaide, Australia; 5 School of Nursing, Hong Kong Polytechnic University, Hung Hom, Hong Kong, Special Administrative Region of China; 6 Impact SRC, Deakin University, Melbourne, Australia; 7 Institute of Clinical Sciences, University of Birmingham, Birmingham, UK

## Abstract

**Background:**

Trial registration helps minimize publication and reporting bias. In leading medical journals, 96% of published trials are registered. The aim of this study was to determine the proportion of randomized controlled trials published in key nursing journals that met criteria for timely registration.

**Methods:**

We reviewed all RCTs published in three (two general, one mental health) nursing journals between August 2011 and September 2016. We classified the included trials as: 1. Not registered, 2. Registered but not reported in manuscript, 3. Registered retrospectively, 4. Registered prospectively (before the recruitment of the first subject into the trial). 5. Timely registration (as 4 but the trial identification number is reported in abstract).

**Results:**

We identified 135 trials published in the three included journals. The majority (*n* = 78, 58%) were not registered. Thirty-three (24%) were retrospectively registered. Of the 24 (18%) trials that were prospectively registered, 11 (8%) met the criteria for timely registration.

**Conclusions:**

There is an unacceptable difference in rates of trial registration between leading medical and nursing journals. Concerted effort is required by nurse researchers, reviewers and journal editors to ensure that all trials are registered in a timely way.

**Electronic supplementary material:**

The online version of this article (doi:10.1186/s41073-017-0036-9) contains supplementary material, which is available to authorized users.

## Background

Estimates indicate that up to 50% of clinical trials are never published, profoundly distorting the evidence base for clinical decision-making [1]. Researchers (and journal editors), while highly motivated to publish findings from trials reporting the effectiveness of a new drug or intervention, tend to be rather less enthusiastic about publishing trials that report negative or inconclusive findings. Registration of trials before the first patient is enrolled goes some way to ensuring that every study is part of the public record and that anyone who is interested can identify whether the data analysis occurred posthoc or outcome measures were amended part way through. In 2004, the International Committee of Medical Journal Editors (ICMJE) issued a statement that from July 2005 all clinical trials published in their journals required registration in a public trial registry [2]. Additionally, the ICMJE issued a policy for Uniform Requirements for Manuscripts (URM) that states that the trial registry identifier must be included at the end of the abstract (http://www.icmje.org/recommendations/browse/publishing-and-editorial-issues/clinical-trial-registration.html).

Authors of a study of 698 trials published in the five ICMJE founding journals (that include the British Medical Journal and the Lancet) reported that 96% of included studies listed a trial identification number [3]. However, only 60% of trials were prospectively registered. These observations suggest that even among leading and well-respected journals there is work to do to ensure timely registration. A study by Harriman and Patel [4] examined rates of prospective and retrospective registration in trials published in the BMC (BioMed Central) series of journals in 2013. They reported that 97% of the 108 published trials were registered. This was completed prospectively in only around a third of cases (*n* = 33, 31%). Scott et al. [5] examined trial registration in five leading psychiatry journals. Of 181 included trials the authors reported that only 60 (33%) were prospectively registered.

Although many nursing journals encourage trial registration, editors, researchers and authors have not spoken with the same clarity and consistency as their medical colleagues, possibly explaining why seemingly few trials published in nursing journals are registered. For example, Jull and Aye [6] reported that of the 83 trials published in the top 15 nursing journals in 2012, only 18 (22%) were registered. The authors report few specific details about registration (for example, the length of registration delay) [6].

In a keynote presentation at the INANE (International Academy of Nursing Editors) conference in London in the summer of 2016 Dr Ben Goldacre—one of the leaders of the alltrials campaign (http://www.alltrials.net)—challenged the assembled audience of nursing journal editors to ensure that trials published in the journals they edit were registered. Inspired by this challenge, we published an editorial [7] in September 2016 where we reported a simple count of the proportion of registered trials published in the *Journal of Advanced Nursing*. We found that the authors of two-thirds of published trials did not to report a registration number.

Reflecting on this observation, we felt there was a need to develop this work further. We reviewed the literature and identified no studies that have examined trial registration practices in key nursing journals. Therefore, we aimed to conduct a study to examine the proportion of RCTs published in three of the key nursing journals, covering both general and mental health nursing that met criteria for timely registration.

## Methods

We decided not to focus on the three top journals listed in the Journal Citation Report (JCR) under the field of nursing (International Journal of Nursing Studies IF (impact factor) = 3.561, Oncology Nursing Forum IF = 2.708, European Journal of Cardiovascular Nursing IF = 2.491) with the highest impact factors. This was because in nursing, unlike in medicine, many of the leading journals have a specialist focus (e.g., oncology, cardiovascular disease). We therefore decided to select what we judged the key general and mental health journals in nursing based on a combination of bibliometric indicators.

The included journals were the *Journal of Advanced Nursing* (*JAN*), the *International Journal of Nursing Studies* (*IJNS*) and the *Journal of Psychiatric and Mental Health Nursing* (*JPM*). Journal Citation Report (total annual citations and IF (ranked for the field, nursing science)), Scopus CiteScore, and GoogleScholar h-5 index for included journals was:

- *JAN* (JCR total citations = 12,952 (rank 1/114) and Impact Factor = 1.917 (ranking 9/114), Scopus CiteScore = 2.29 (rank 5/91 for nursing), GoogleScholar h5-index = 51 (ranked second for nursing).
- *IJNS* (JCR total citations = 5,898 (ranking 3/114) and Impact Factor = 3.561 (ranking 1/114), Scopus CiteScore = 3.49 (rank 2/91 for nursing), GoogleScholar h5-index = 53 (ranked first for nursing).
- *JPM* (JCR total citation = 1,956 (rank 14/114) and Impact Factor = 1.055 (ranking 56/114 for nursing science), Scopus CiteScore = 1.26 (rank 8/38 for psychiatric mental health), GoogleScholar h5-index = 27 (ranked 21st for nursing).

*JAN* author guidelines state that trials “should be registered publicly”; the *IJNS* “encourages” trial registration. The *JPM* requires that authors submit a CONSORT (Consolidated Standards for Reporting Randomized Controlled Trials) checklist, where item 1b states the registration number should be included [8].

We extracted all randomized clinical trials published in included journals between the 1 September 2011 and 31 August 2016. For this study, we used the WHO [9] definition of a clinical trial:

“A clinical trial is any research study that prospectively assigns human participants or groups of humans to one or more health-related interventions to evaluate the effects on health outcomes.”

We excluded trials where participants were not randomized (e.g., quasi-experimental studies). We did this as there has been some debate among nursing editors about the need for non-randomized studies to be registered [10]. Two researchers independently read the title and abstract of every paper published in included journals looking for the phrases “randomized controlled,” “pilot”, “feasibility”, “stepped wedge”, or “clinical trial.” A five-year window was selected so that we could explore trends in rates of registration. Full texts of manuscripts were then retrieved and checked to ensure that the trial reported met inclusion criteria. We excluded studies that were not randomized, recruited subjects before 1 July 2005, or reported secondary analysis from a previously published trial. From included studies, researchers extracted the following information:

- Name of publishing journal
- The trial registration number
- Trial registration number in the manuscript abstract
- If no trial registration number was reported (in either the abstract or the body of the text), we manually checked CTG, ISRCTN and the trial registry for the country of the corresponding author using keywords from the trial. Additionally, we also contacted the corresponding author by email to check if the trial was registered. We did this to assess if the trial had been registered but not reported.
- Design (e.g., RCT, cluster RCT, stepped wedged, pilot, feasibility)
- The country of affiliation given of the corresponding author
- Presence of a conflict of interest statement

The trial registration number was used to extract the following information from the relevant registry:

- The name of the registry
- The date the trial was registered
- The date the first subject was enrolled in the study

Four researchers (a combination of AB, EH, DT, and LB) completed data extraction independently using a form we devised for the study. Two team members reviewed each paper. Coding discrepancies were discussed with a senior researcher (RG) who arbitrated a final decision. We uploaded extracted data into SPSS that was used to classify, describe, and analyze the data. We classified included studies into five groups:

1. Not registered: the trial authors provided no information about trial registration in the published manuscript.
2. Registered but not reported: the trial was registered but the registration number was not reported by the authors anywhere in the manuscript.
3. Retrospectively registered: the time of trial registration was after the date of first patient enrollment into the trial.
4. Prospectively registered: the time of trial registration preceded the date the first patient was enrolled into the study.
5. Timely registration: as 4, but the papers abstract contains the trial registration number (a ICMJE URM requirement).

As in the Harriman and Patel [4] study, we also calculated the length of registration delay. We defined this as the number of days between the date of first patient enrolment and the date of trial registration. We reported the median number of days and IQR (interquartile range) of delay for each journal.

We defined the date of trial registration as the date the registration number was issued. If this date was not stated, then we used the date of application to the registry. There are some inconsistencies across registries about how study start dates are recorded. Where possible, we intended to extract the actual date the first patient was recruited. We were aware this would not always be possible. In instances where only the study start date or anticipated start date were given, this was used as a proxy for the date of first patient enrollment. If the month/year but not the day of recruitment was recorded in the database, then we assumed enrollment began on the first of the month.

## Results

**Fig. 1:**
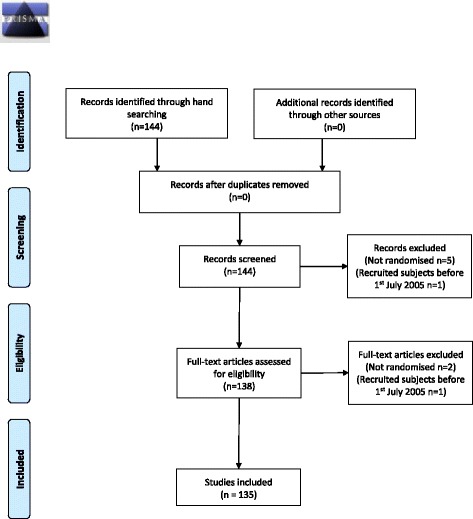
PRISMA 2009 flow diagram

Figure 1 shows the flow of papers through this study. We identified 135 RCTs that met inclusion criteria (Table 1). These represented 5% (*n* = 135/2852) of the papers published in the three journals over the study period (*JAN n* = 42/1350, 3%; *IJNS n* = 80/940, 9%; *JPM n* = 13/562, 2%). Most of the studies were described as RCTs; there were a small number of cluster (*n* = 11), pilot (*n* = 5), feasibility (*n* = 2) and stepped wedge (*n* = 1) trials. The majority (83/135, 62%) of trials were not registered. From checking trial registries and contacting authors of unregistered studies directly we identified three studies (3/78, 4%) that had been registered but registration was not reported in the manuscript. Ten percent of trials were prospectively registered (*n* = 13/135) and eight percent were prospectively registered with the trial identification number in the abstract (timely registration) (*n* = 11/135).

**Table 1:** Number ( %) of trials registered

	Not registered (*n* = 78)	Registered but not reported (*n* = 3)	Retrospectively registered (*n* = 30)	Prospectively registered (*n* = 13)	Prospectively registered (ID in abstract) (*n* = 11)	Total
Journal						
*IJNS*	40 (50%)	3 (4%)	20 (25%)	10 (12%)	7 (9%)	80
*JAN*	28 (67%)	-	7 (17%)	3 (7%)	4 (10%)	42
*JPM*	10 (77%)	-	3 (23%)	-	-	13
Trial registry						
Clinicaltrial.gov^a^	-	2 (6%)	16 (52%)	5 (16%)	8 (26%)	31
ISRCTN^b^	-	-	6 (60%)	3 (30%)	1 (10%)	10
ANZCTR^c^	-	1 (11%)	6 (67%)	-	2 (22%)	9
HKRCTN^d^	-	-	-	2 (100%)	-	2
Iranian trail registry^e^	-	-	1 (100%)	-	-	1
Chinese trial registry^f^	-	-	1 (100%)	-	-	1
NTR^g^	-	-	0 (0%)	3 (100%)	-	3
Design						
RCT	75 (64%)	2 (2%)	24 (20%)	10 (8%)	7 (6%)	118
Cluster RCT	1 (9%)	1 (9%)	3 (27%)	3 (27%)	3 (27%)	11
Stepped wedge	1 (100%)	-	-	-	-	1
Pilot	3 (60%)	-	2 (40%)	-	-	5
Feasibility study	-	-	1 (50%)	-	1 (50%)	2
Continent of corresponding author affiliation						
Asia	53 (71%)	2 (3%)	17 (23%)	2 (3%)	1 (1%)	75
Australia and NZ	3 (38%)	1 (13%)	3 (38%)	-	1 (13%)	8
Europe	17 (40%)	-	5 (16%)	11 (27%)	7 (17%)	40
North and South America	7 (54%)	-	5 (36%)	-	2 (14%)	14
Conflicts of interests						
Declared	71 (57%)	3 (2%)	28 (23%)	13 (10%)	10 (8%)	125
Not declared	9 (75%)	-	2 (17%)	-	1 (8%)	12

^a^Clinicaltrials.gov ^b^ISRCTN.org ^c^Australian New Zealand Clinical Trial Registry ^d^Hong Kong (not a WHO primary registry as of the 30th November 2016) ^e^Iranian Clinical Trials Registry ^f^Chinese Clinical Trial Registry ^g^The Netherland National Trial Registry

Around a quarter of trials (*n* = 33/135, 24%) were registered retrospectively, the length of delay was between 39 and 2,463 days and the median was 675 (IQR 563–1099) days (*JAN* median 1043, IQR 654–1074; *IJNS* median 778, IQR 241–1410). We were not able to calculate the median or IQR for *JPM* as so few studies were registered.

The most commonly used trial registry was CTG (clinicaltrials.gov) (*n* = 31/57, 54%). Trial (corresponding) authors came from 30 different countries, over half (*n* = 75/135, 56%) were from Asia. We observed that corresponding authors from Asia were less likely to have registered their trial compared with those from the rest of the world. A post hoc analysis suggested this association was statistically significant (OR = .30, 95% CI .15, .61, *p* = 001).

The majority of study authors provided a conflicts of interest statement (*n* = 125/135, 93%), typically authors reported no competing interests. Table 2 shows the proportion of studies registered in each of the 5 years. Over the study period, we did not observe an obvious increase in the number of trials published. There was also no apparent trend suggesting an increase in the proportion of trials registered.

**Table 2:** Number of RCTs registered over the 5-year study period

Year	Date	Total number of included trials	Number of trials not registered (*n*, %)
1	1 September 2011 to 31 August 2012	32	22 (69%)
2	1 September 2012 to 31 August 2013	22	11 (50%)
3	1 September 2013 to 31 August 2014	28	17 (61%)
4	1 September 2014 to 31 August 2015	34	17 (50%)
5	1 September 2015 to 31 August 2016	19	11 (58%)

## Discussion

The aim of this study was to determine the proportion of RCTs published in key nursing journals that met criteria for timely registration. As might be expected from a maturing academic discipline that includes a broad research constituency, RCTs made up only a modest proportion of the manuscripts published. Nevertheless, few included trials were registered. Our observation is slightly more positive but broadly consistent with Jull and Aye [6] who reported that 22% of trials published in 15 nursing journals in 2012 were registered. This may suggest that registration rates in the key journals we included are higher than in nursing journals more generally.

Rates of registration in this study differed markedly to those in trials published in medical journals. For example, Harriman and Patel [4] and Huser and Cimino [3], respectively, reported that only 3% of trials in BMC journals and a similar proportion in ICMJE founding journals were not registered. Lower rates of trial registration have been reported in specific areas of medicine; for example, in a study of ten high impact surgical journals, 35% of trials were not registered [11]. There was a marked difference in the rates of prospective registration between *JPM* and the top psychiatry journals (0 vs. 33%) [5], though there are comparatively very few trials published in mental health nursing journals.

Possible explanations as to why registration rates were low might include administrative delay in registration, author awareness, and ambiguous journal author guidelines. There were no studies where registration occurred within one month of the trial starting, suggesting that it was not administrative issues that explained low registration rates. Instead, our findings may highlight a potential lack of awareness of the importance of prospective registration, reflecting that this is an emerging topic for discussion in nursing research. This seems to be particurarly profound among nurse investigators working in Asia. A study by Reveiz et al. [12] has reported low levels of support among trialists for registration. We were unable to identify any published studies that have examined the views of nurse researchers about the importance of trial registration; exploring their attitudes in more detail would be illuminating for further research.

The ICMJE statement explicitly *requires* trial registration. Two included journals broadly endorse registration (*JAN and IJNS*). *JPM* requires authors submit a CONSORT checklist, effectively ensuring that registration is required. There remains a need to develop a greater awareness among nurse authors, reviewers, and editors of the importance of trial registration. In our view, all nursing journal editors need to be explicit about the need for authors to register trials.

The authors of three trials had registered their study but not reported that they had done so in the published manuscript. We note that for all three manuscripts registration was retrospective. It is concerning that a small number of authors had gone to the effort of registering their study but not felt that it is sufficiently important to report that they have done this.

### The ethics of retrospective registration

In this study, if trials were registered, this was most likely done retrospectively. Boughton [13] has discussed the ethical dilemma of accepting retrospectively registered trials for publication. On the one hand, considering a retrospectively registered paper for publication undermines the aim of trial registration and some authors have argued that the value of retrospective registration in countering publication or selective reporting bias is negligible [14, 15]. On the other hand, there is an ethical responsibility to publish research, given the investment subjects have made in volunteering to participate.

### The Asia powerhouse

It is interesting to observe that the corresponding author for the majority of included trials was from Asia. By contrast, most trials published in BMC journals were from Europe. Our observations seem to suggest that Asia is a powerhouse in nursing trials research, at least regarding work published in some of the profession’s key journals. That rates of registration are seemingly lower among this group of authors is of concern and indicates that work may be required to increase awareness in a global sense, given the different compliance patterns identified.

### Limitations

In this study, our denominator was the total number of published randomized controlled trials. We excluded non-RCTs (e.g., controlled clinical trials) as there continues to be some debate about whether registration is required [10]. Arguably, we could have been more inclusive in the types of trials we included. The consequence may be that we have overestimated rates of trial registration.

We extracted the study start date from the trial registry record. Some manuscripts may also have included details of trial start dates. We did not check if registry/manuscript dates matched. It is possible that some start dates recorded in trial registries were inaccurate, i.e., that the trial started later than reported. This may mean that we had incorrectly classified some trials as retrospectively registered when in fact they were prospectively registered. However, we assert that it is the responsibility of the research team to ensure that their trial registry entry is up to date and correct. We would recommend that in future studies of this type, both registry and manuscript recorded start dates are extracted, checked, and any inconsistencies resolved with the trial corresponding author.

In this study, we did not compare registered to published outcomes or outcome classification (e.g., primary, secondary). Previous authors have observed that registered and published outcomes are often different [14]. This is a major limitation and we would recommend that future research should address this deficit.

We have incorporated a list of included papers as a supplementary document (Additional file 1). The classification we assigned to each study is not included. This may be considered a limitation of our study. However, we were concerned that publishing our classifications may lead to a perception of “naming and shaming” those who had not registered their trials. We feel that such a response would be counter to our intention in conducting this study that was primarily to raise awareness of the need for timely prospective registration of trials by nursing researchers and authors. It may be that authors of future studies could consider including these data in their reporting.

Our current study is, of course, a snapshot of three journals, and we cannot be confident that our observations are generalizable to all nursing journals.

## Conclusions

Our study draws attention to important shortcomings in the reporting of clinical trials in nursing journals. There are over a hundred journals (*n* = 114) listed in the JCR under the field of nursing (science). In this study, we focused on just three, which are, arguably, key general/mental health journals in the field. It is important that all nursing journals have a clear and explicit policy requiring registration of trial they publish. However, we recognize that enforcing these policies is challenging. Editors clearly have an important role to play, but ultimately there is no real incentive for them to mandate registration. Perhaps, as in medicine, editors of leading journals need to work collaboratively and to ensure that they lead by example.

## Additional file

Additional file 1: Included studies. (DOCX 31 kb)

## Abbreviations

BMC: BioMed Central
CONSORT: Consolidated Standards for Reporting Randomized Controlled Trials
CTG: Clinicaltrials.gov
ICMJE: International Committee of Mental Journal Editors
ID: Identification
IJNS: International Journal of Nursing Studies
IQR: Interquartile range
JAN: Journal of Advanced Nursing
JPM: Journal of Psychiatric and Mental Health Nursing
RCT: Randomised Controlled Trial
URM: Uniform Requirements for Manuscripts
WHO: World Health Organization
