# Supplementary material for: Registration of randomized controlled trials in nursing journals
Source: Res Integr Peer Rev. 2017 Jul 16;2:8. doi: 10.1186/s41073-017-0036-9 (PMC5803636; doi:10.1186/s41073-017-0036-9)
Supplement: Additional file 1: — Included studies. (DOCX 31 kb) [file 41073_2017_36_MOESM1_ESM.docx]

**Included study reference list**

Aghaie, B., Rejeh, N., Heravi-Karimooi, M., Ebadi, A., Moradian, S. T., Vaismoradi, M., & Jasper, M. (2014). Effect of nature-based sound therapy on agitation and anxiety in coronary artery bypass graft patients during the weaning of mechanical ventilation: A randomised clinical trial. *International Journal of Nursing Studies*, *51*(4), 526–538. https://doi.org/10.1016/j.ijnurstu.2013.08.003

Allen, V. J., Methven, L., & Gosney, M. (2014). Impact of serving method on the consumption of nutritional supplement drinks: randomized trial in older adults with cognitive impairment. *Journal of Advanced Nursing*, *70*(6), 1323–1333. https://doi.org/10.1111/jan.12293

Ang, E., Mordiffi, S. Z., & Wong, H. B. (2011). Evaluating the use of a targeted multiple intervention strategy in reducing patient falls in an acute care hospital: a randomized controlled trial. *Journal of Advanced Nursing*, *67*(9), 1984–1992. https://doi.org/10.1111/j.1365-2648.2011.05646.x

Arslan-Özkan, İ., Okumuş, H., & Buldukoğlu, K. (2014). A randomized controlled trial of the effects of nursing care based on Watson’s Theory of Human Caring on distress, self-efficacy and adjustment in infertile women. *Journal of Advanced Nursing*, *70*(8), 1801–1812. https://doi.org/10.1111/jan.12338

Arts, E. E. A., Landewe-Cleuren, S. A. N. T., Schaper, N. C., & Vrijhoef, H. J. M. (2012). The cost-effectiveness of substituting physicians with diabetes nurse specialists: a randomized controlled trial with 2-year follow-up. *Journal of Advanced Nursing*, *68*(6), 1224–1234. https://doi.org/10.1111/j.1365-2648.2011.05797.x

Arvidsson, S., Bergman, S., Arvidsson, B., Fridlund, B., & Tingström, P. (2013). Effects of a self-care promoting problem-based learning programme in people with rheumatic diseases: a randomized controlled study. *Journal of Advanced Nursing*, *69*(7), 1500–1514. https://doi.org/10.1111/jan.12008

Beeckman, D., Clays, E., Van Hecke, A., Vanderwee, K., Schoonhoven, L., & Verhaeghe, S. (2013). A multi-faceted tailored strategy to implement an electronic clinical decision support system for pressure ulcer prevention in nursing homes: a two-armed randomized controlled trial. *International Journal of Nursing Studies*, *50*(4), 475–486. https://doi.org/10.1016/j.ijnurstu.2012.09.007

Bowers, L., James, K., Quirk, A., Simpson, A., SUGAR, Stewart, D., & Hodsoll, J. (2015). Reducing conflict and containment rates on acute psychiatric wards: The Safewards cluster randomised controlled trial. *International Journal of Nursing Studies*, *52*(9), 1412–1422. https://doi.org/10.1016/j.ijnurstu.2015.05.001

Calvo, R., Martínez-Zapata, M. J., Urrútia, G., Gich, I., Jordán, M., Del Arco, A., … Bonfill, X. (2012). Low- vs. high-pressure suction drainage after total knee arthroplasty: a double-blind randomized controlled trial. *Journal of Advanced Nursing*, *68*(4), 758–766. https://doi.org/10.1111/j.1365-2648.2011.05760.x

Champion, J. D., & Collins, J. L. (2012). Comparison of a theory-based (AIDS Risk Reduction Model) cognitive behavioral intervention versus enhanced counseling for abused ethnic minority adolescent women on infection with sexually transmitted infection: results of a randomized controlled trial. *International Journal of Nursing Studies*, *49*(2), 138–150. https://doi.org/10.1016/j.ijnurstu.2011.08.010

Chan, S. S. C., Leung, D. Y. P., Leung, A. Y. M., Lam, C., Hung, I., Chu, D., … Yuen, K. Y. (2015). A nurse-delivered brief health education intervention to improve pneumococcal vaccination rate among older patients with chronic diseases: a cluster randomized controlled trial. *International Journal of Nursing Studies*, *52*(1), 317–324. https://doi.org/10.1016/j.ijnurstu.2014.06.008

Chang, E.-T., Lai, H.-L., Chen, P.-W., Hsieh, Y.-M., & Lee, L.-H. (2012). The effects of music on the sleep quality of adults with chronic insomnia using evidence from polysomnographic and self-reported analysis: a randomized control trial. *International Journal of Nursing Studies*, *49*(8), 921–930. https://doi.org/10.1016/j.ijnurstu.2012.02.019

Chang, S.-M., & Chen, C.-H. (2016). Effects of an intervention with drinking chamomile tea on sleep quality and depression in sleep disturbed postnatal women: a randomized controlled trial. *Journal of Advanced Nursing*, *72*(2), 306–315. https://doi.org/10.1111/jan.12836

Chen, J.-H., Chao, Y.-H., Lu, S.-F., Shiung, T.-F., & Chao, Y.-F. (2012). The effectiveness of valerian acupressure on the sleep of ICU patients: a randomized clinical trial. *International Journal of Nursing Studies*, *49*(8), 913–920. https://doi.org/10.1016/j.ijnurstu.2012.02.012

Chen, K.-M., Li, C.-H., Chang, Y.-H., Huang, H.-T., & Cheng, Y.-Y. (2015). An elastic band exercise program for older adults using wheelchairs in Taiwan nursing homes: a cluster randomized trial. *International Journal of Nursing Studies*, *52*(1), 30–38. https://doi.org/10.1016/j.ijnurstu.2014.06.005

Chen, K.-M., Li, C.-H., Huang, H.-T., & Cheng, Y.-Y. (2016). Feasible modalities and long-term effects of elastic band exercises in nursing home older adults in wheelchairs: A cluster randomized controlled trial. *International Journal of Nursing Studies*, *55*, 4–14. https://doi.org/10.1016/j.ijnurstu.2015.11.004

Chen, M.-C., Liu, H.-E., Huang, H.-Y., & Chiou, A.-F. (2012). The effect of a simple traditional exercise programme (Baduanjin exercise) on sleep quality of older adults: a randomized controlled trial. *International Journal of Nursing Studies*, *49*(3), 265–273. https://doi.org/10.1016/j.ijnurstu.2011.09.009

Chen, S. M., Creedy, D., Lin, H.-S., & Wollin, J. (2012). Effects of motivational interviewing intervention on self-management, psychological and glycemic outcomes in type 2 diabetes: a randomized controlled trial. *International Journal of Nursing Studies*, *49*(6), 637–644. https://doi.org/10.1016/j.ijnurstu.2011.11.011

Cheng, C.-M., Chiu, M.-J., Wang, J.-H., Liu, H.-C., Shyu, Y.-I. L., Huang, G.-H., & Chen, C. C.-H. (2012). Cognitive stimulation during hospitalization improves global cognition of older Taiwanese undergoing elective total knee and hip replacement surgery. *Journal of Advanced Nursing*, *68*(6), 1322–1329. https://doi.org/10.1111/j.1365-2648.2011.05842.x

Chien, W. T., & Chan, S. W. C. (2013). The effectiveness of mutual support group intervention for Chinese families of people with schizophrenia: a randomised controlled trial with 24-month follow-up. *International Journal of Nursing Studies*, *50*(10), 1326–1340. https://doi.org/10.1016/j.ijnurstu.2013.01.004

Chien, W. T., Yip, A. L. K., Liu, J. Y. W., & McMaster, T. W. (2016). The effectiveness of manual-guided, problem-solving-based self-learning programme for family caregivers of people with recent-onset psychosis: A randomised controlled trial with 6-month follow-up. *International Journal of Nursing Studies*, *59*, 141–155. https://doi.org/10.1016/j.ijnurstu.2016.03.018

Chow, M. C. M., Kwok, S.-M., Luk, H.-W., Law, J. W. H., & Leung, B. P. K. (2012). Effect of continuous oral suctioning on the development of ventilator-associated pneumonia: a pilot randomized controlled trial. *International Journal of Nursing Studies*, *49*(11), 1333–1341. https://doi.org/10.1016/j.ijnurstu.2012.06.003

Chow, S. K. Y., & Wong, F. K. Y. (2014). A randomized controlled trial of a nurse-led case management programme for hospital-discharged older adults with co-morbidities. *Journal of Advanced Nursing*, *70*(10), 2257–2271. https://doi.org/10.1111/jan.12375

Chuang, L.-L., Lin, L.-C., Cheng, P.-J., Chen, C.-H., Wu, S.-C., & Chang, C.-L. (2012a). Effects of a relaxation training programme on immediate and prolonged stress responses in women with preterm labour. *Journal of Advanced Nursing*, *68*(1), 170–180. https://doi.org/10.1111/j.1365-2648.2011.05765.x

Chuang, L.-L., Lin, L.-C., Cheng, P.-J., Chen, C.-H., Wu, S.-C., & Chang, C.-L. (2012b). The effectiveness of a relaxation training program for women with preterm labour on pregnancy outcomes: a controlled clinical trial. *International Journal of Nursing Studies*, *49*(3), 257–264. https://doi.org/10.1016/j.ijnurstu.2011.09.007

Cicolini, G., Simonetti, V., Comparcini, D., Celiberti, I., Di Nicola, M., Capasso, L. M., … Manzoli, L. (2014). Efficacy of a nurse-led email reminder program for cardiovascular prevention risk reduction in hypertensive patients: a randomized controlled trial. *International Journal of Nursing Studies*, *51*(6), 833–843. https://doi.org/10.1016/j.ijnurstu.2013.10.010

Cockayne, S., Pattenden, J., Worthy, G., Richardson, G., & Lewin, R. (2014). Nurse facilitated Self-management support for people with heart failure and their family carers (SEMAPHFOR): a randomised controlled trial. *International Journal of Nursing Studies*, *51*(9), 1207–1213. https://doi.org/10.1016/j.ijnurstu.2014.01.010

Cossette, S., Frasure-Smith, N., Vadeboncoeur, A., McCusker, J., & Guertin, M.-C. (2015). The impact of an emergency department nursing intervention on continuity of care, self-care capacities and psychological symptoms: secondary outcomes of a randomized controlled trial. *International Journal of Nursing Studies*, *52*(3), 666–676. https://doi.org/10.1016/j.ijnurstu.2014.12.007

Craft, M. A., Davis, G. C., & Paulson, R. M. (2013). Expressive writing in early breast cancer survivors. *Journal of Advanced Nursing*, *69*(2), 305–315. https://doi.org/10.1111/j.1365-2648.2012.06008.x

Crowe, M., Inder, M., Carlyle, D., Wilson, L., Whitehead, L., Panckhurst, A., … Joyce, P. (2012). Nurse-led delivery of specialist supportive care for bipolar disorder: a randomized controlled trial. *Journal of Psychiatric and Mental Health Nursing*, *19*(5), 446–454. https://doi.org/10.1111/j.1365-2850.2011.01822.x

de Jong, M., Lucas, C., Bredero, H., van Adrichem, L., Tibboel, D., & van Dijk, M. (2012). Does postoperative “M” technique massage with or without mandarin oil reduce infants’ distress after major craniofacial surgery? *Journal of Advanced Nursing*, *68*(8), 1748–1757. https://doi.org/10.1111/j.1365-2648.2011.05861.x

Demarré, L., Beeckman, D., Vanderwee, K., Defloor, T., Grypdonck, M., & Verhaeghe, S. (2012). Multi-stage versus single-stage inflation and deflation cycle for alternating low pressure air mattresses to prevent pressure ulcers in hospitalised patients: a randomised-controlled clinical trial. *International Journal of Nursing Studies*, *49*(4), 416–426. https://doi.org/10.1016/j.ijnurstu.2011.10.007

Ezenwa, M. O., Yao, Y., Engeland, C. G., Molokie, R. E., Wang, Z. J., Suarez, M. L., & Wilkie, D. J. (2016). A randomized controlled pilot study feasibility of a tablet-based guided audio-visual relaxation intervention for reducing stress and pain in adults with sickle cell disease. *Journal of Advanced Nursing*, *72*(6), 1452–1463. https://doi.org/10.1111/jan.12895

Fallahi Khoshknab, M., Sheikhona, M., Rahgouy, A., Rahgozar, M., & Sodagari, F. (2014). The effects of group psychoeducational programme on family burden in caregivers of Iranian patients with schizophrenia. *Journal of Psychiatric and Mental Health Nursing*, *21*(5), 438–446. https://doi.org/10.1111/jpm.12107

Fletcher, S. J., Waterman, H., Nelson, L., Carter, L. A., Dwyer, L., Roberts, C., … Kitchener, H. (2015). Holistic assessment of women with hyperemesis gravidarum: A randomised controlled trial. *International Journal of Nursing Studies*, *52*(11), 1669–1677. https://doi.org/10.1016/j.ijnurstu.2015.06.007

Furuya, R. K., Arantes, E. C., Dessotte, C. A. M., Ciol, M. A., Hoffman, J. M., Schmidt, A., … Rossi, L. A. (2015). A randomized controlled trial of an educational programme to improve self-care in Brazilian patients following percutaneous coronary intervention. *Journal of Advanced Nursing*, *71*(4), 895–908. https://doi.org/10.1111/jan.12568

Furze, G., Cox, H., Morton, V., Chuang, L.-H., Lewin, R. J. P., Nelson, P., … Elton, P. (2012). Randomized controlled trial of a lay-facilitated angina management programme. *Journal of Advanced Nursing*, *68*(10), 2267–2279. https://doi.org/10.1111/j.1365-2648.2011.05920.x

Gao, H., Xu, G., Gao, H., Dong, R., Fu, H., Wang, D., … Zhang, H. (2015). Effect of repeated Kangaroo Mother Care on repeated procedural pain in preterm infants: A randomized controlled trial. *International Journal of Nursing Studies*, *52*(7), 1157–1165. https://doi.org/10.1016/j.ijnurstu.2015.04.006

Gao, L., Xie, W., Yang, X., & Chan, S. W. (2015). Effects of an interpersonal-psychotherapy-oriented postnatal programme for Chinese first-time mothers: a randomized controlled trial. *International Journal of Nursing Studies*, *52*(1), 22–29. https://doi.org/10.1016/j.ijnurstu.2014.06.006

Gao, L.-L., Chan, S. W.-C., & Sun, K. (2012). Effects of an interpersonal-psychotherapy-oriented childbirth education programme for Chinese first-time childbearing women at 3-month follow up: randomised controlled trial. *International Journal of Nursing Studies*, *49*(3), 274–281. https://doi.org/10.1016/j.ijnurstu.2011.09.010

Gómez-Urquiza, J. L., Hueso-Montoro, C., Urquiza-Olmo, J., Ibarrondo-Crespo, R., González-Jiménez, E., & Schmidt-Riovalle, J. (2016). A randomized controlled trial of the effect of a photographic display with and without music on pre-operative anxiety. *Journal of Advanced Nursing*, *72*(7), 1666–1676. https://doi.org/10.1111/jan.12937

Gonge, H., & Buus, N. (2015). Is it possible to strengthen psychiatric nursing staff’s clinical supervision? RCT of a meta-supervision intervention. *Journal of Advanced Nursing*, *71*(4), 909–921. https://doi.org/10.1111/jan.12569

Gouveia, B. R., Gonçalves Jardim, H., Martins, M. M., Gouveia, É. R., de Freitas, D. L., Maia, J. A., & Rose, D. J. (2016). An evaluation of a nurse-led rehabilitation programme (the ProBalance Programme) to improve balance and reduce fall risk of community-dwelling older people: A randomised controlled trial. *International Journal of Nursing Studies*, *56*, 1–8. https://doi.org/10.1016/j.ijnurstu.2015.12.004

Gu, C., Wu, X., Ding, Y., Zhu, X., & Zhang, Z. (2013). The effectiveness of a Chinese midwives’ antenatal clinic service on childbirth outcomes for primipare: a randomised controlled trial. *International Journal of Nursing Studies*, *50*(12), 1689–1697. https://doi.org/10.1016/j.ijnurstu.2013.05.001

Gudmundsdottir, R. M., & Thome, M. (2014). Evaluation of the effects of individual and group cognitive behavioural therapy and of psychiatric rehabilitation on hopelessness of depressed adults: a comparative analysis. *Journal of Psychiatric and Mental Health Nursing*, *21*(10), 866–872. https://doi.org/10.1111/jpm.12157

Guo, P., East, L., & Arthur, A. (2012). A preoperative education intervention to reduce anxiety and improve recovery among Chinese cardiac patients: a randomized controlled trial. *International Journal of Nursing Studies*, *49*(2), 129–137. https://doi.org/10.1016/j.ijnurstu.2011.08.008

Hälleberg Nyman, M., Gustafsson, M., Langius-Eklöf, A., Johansson, J.-E., Norlin, R., & Hagberg, L. (2013). Intermittent versus indwelling urinary catheterisation in hip surgery patients: a randomised controlled trial with cost-effectiveness analysis. *International Journal of Nursing Studies*, *50*(12), 1589–1598. https://doi.org/10.1016/j.ijnurstu.2013.05.007

He, H.-G., Zhu, L., Chan, S. W.-C., Liam, J. L. W., Li, H. C. W., Ko, S. S., … Wang, W. (2015). Therapeutic play intervention on children’s perioperative anxiety, negative emotional manifestation and postoperative pain: a randomized controlled trial. *Journal of Advanced Nursing*, *71*(5), 1032–1043. https://doi.org/10.1111/jan.12608

He, H.-G., Zhu, L.-X., Chan, W.-C. S., Liam, J. L. W., Ko, S. S., Li, H. C. W., … Yobas, P. (2015). A mixed-method study of effects of a therapeutic play intervention for children on parental anxiety and parents’ perceptions of the intervention. *Journal of Advanced Nursing*, *71*(7), 1539–1551. https://doi.org/10.1111/jan.12623

Heinen, M., Borm, G., van der Vleuten, C., Evers, A., Oostendorp, R., & van Achterberg, T. (2012). The Lively Legs self-management programme increased physical activity and reduced wound days in leg ulcer patients: Results from a randomized controlled trial. *International Journal of Nursing Studies*, *49*(2), 151–161. https://doi.org/10.1016/j.ijnurstu.2011.09.005

Hmwe, N. T. T., Subramanian, P., Tan, L. P., & Chong, W. K. (2015). The effects of acupressure on depression, anxiety and stress in patients with hemodialysis: a randomized controlled trial. *International Journal of Nursing Studies*, *52*(2), 509–518. https://doi.org/10.1016/j.ijnurstu.2014.11.002

Huang, T.-T., Liu, C.-B., Tsai, Y.-H., Chin, Y.-F., & Wong, C.-H. (2015). Physical fitness exercise versus cognitive behavior therapy on reducing the depressive symptoms among community-dwelling elderly adults: A randomized controlled trial. *International Journal of Nursing Studies*, *52*(10), 1542–1552. https://doi.org/10.1016/j.ijnurstu.2015.05.013

Huang, Y.-C., Chen, H.-H., Yeh, M.-L., & Chung, Y.-C. (2012). Case studies combined with or without concept maps improve critical thinking in hospital-based nurses: a randomized-controlled trial. *International Journal of Nursing Studies*, *49*(6), 747–754. https://doi.org/10.1016/j.ijnurstu.2012.01.008

Hudson, B. F., Davidson, J., & Whiteley, M. S. (2015). The impact of hand reflexology on pain, anxiety and satisfaction during minimally invasive surgery under local anaesthetic: a randomised controlled trial. *International Journal of Nursing Studies*, *52*(12), 1789–1797. https://doi.org/10.1016/j.ijnurstu.2015.07.009

Huis, A., Hulscher, M., Adang, E., Grol, R., van Achterberg, T., & Schoonhoven, L. (2013). Cost-effectiveness of a team and leaders-directed strategy to improve nurses’ adherence to hand hygiene guidelines: a cluster randomised trial. *International Journal of Nursing Studies*, *50*(4), 518–526. https://doi.org/10.1016/j.ijnurstu.2012.11.016

Huis, A., Schoonhoven, L., Grol, R., Donders, R., Hulscher, M., & van Achterberg, T. (2013). Impact of a team and leaders-directed strategy to improve nurses’ adherence to hand hygiene guidelines: a cluster randomised trial. *International Journal of Nursing Studies*, *50*(4), 464–474. https://doi.org/10.1016/j.ijnurstu.2012.08.004

Iglesias, B., Ramos, F., Serrano, B., Fàbregas, M., Sánchez, C., García, M. J., … PIPA Group. (2013). A randomized controlled trial of nurses vs. doctors in the resolution of acute disease of low complexity in primary care. *Journal of Advanced Nursing*, *69*(11), 2446–2457. https://doi.org/10.1111/jan.12120

Jeon, Y.-H., Luscombe, G., Chenoweth, L., Stein-Parbury, J., Brodaty, H., King, M., & Haas, M. (2012). Staff outcomes from the caring for aged dementia care resident study (CADRES): a cluster randomised trial. *International Journal of Nursing Studies*, *49*(5), 508–518. https://doi.org/10.1016/j.ijnurstu.2011.10.020

Jonsdottir, H., Amundadottir, O. R., Gudmundsson, G., Halldorsdottir, B. S., Hrafnkelsson, B., Ingadottir, T. S., … Stefansdottir, I. K. (2015). Effectiveness of a partnership-based self-management programme for patients with mild and moderate chronic obstructive pulmonary disease: a pragmatic randomized controlled trial. *Journal of Advanced Nursing*, *71*(11), 2634–2649. https://doi.org/10.1111/jan.12728

Kahraman, B. B., & Ozdemir, L. (2015). The impact of abdominal massage administered to intubated and enterally fed patients on the development of ventilator-associated pneumonia: a randomized controlled study. *International Journal of Nursing Studies*, *52*(2), 519–524. https://doi.org/10.1016/j.ijnurstu.2014.11.001

Kassab, M., Sheehy, A., King, M., Fowler, C., & Foureur, M. (2012). A double-blind randomised controlled trial of 25% oral glucose for pain relief in 2-month old infants undergoing immunisation. *International Journal of Nursing Studies*, *49*(3), 249–256. https://doi.org/10.1016/j.ijnurstu.2011.09.013

Kelechi, T. J., Mueller, M., Zapka, J. G., & King, D. E. (2011). The effect of a cryotherapy gel wrap on the microcirculation of skin affected by chronic venous disorders. *Journal of Advanced Nursing*, *67*(11), 2337–2349. https://doi.org/10.1111/j.1365-2648.2011.05680.x

Kellett, S., Wilbram, M., Davis, C., & Hardy, G. (2014). Team consultancy using cognitive analytic therapy: a controlled study in assertive outreach. *Journal of Psychiatric and Mental Health Nursing*, *21*(8), 687–697. https://doi.org/10.1111/jpm.12123

Khazaee-Pool, M., Sadeghi, R., Majlessi, F., & Rahimi Foroushani, A. (2015). Effects of physical exercise programme on happiness among older people. *Journal of Psychiatric and Mental Health Nursing*, *22*(1), 47–57. https://doi.org/10.1111/jpm.12168

Khresheh, R., Suhaimat, A., Jalamdeh, F., & Barclay, L. (2011). The effect of a postnatal education and support program on breastfeeding among primiparous women: a randomized controlled trial. *International Journal of Nursing Studies*, *48*(9), 1058–1065. https://doi.org/10.1016/j.ijnurstu.2011.02.001

Kim, H., Yoshida, H., & Suzuki, T. (2011). The effects of multidimensional exercise treatment on community-dwelling elderly Japanese women with stress, urge, and mixed urinary incontinence: a randomized controlled trial. *International Journal of Nursing Studies*, *48*(10), 1165–1172. https://doi.org/10.1016/j.ijnurstu.2011.02.016

Kim, M., & Yoon, H. (2011). Comparison of post-dural puncture headache and low back pain between 23 and 25 gauge Quincke spinal needles in patients over 60 years: randomized, double-blind controlled trial. *International Journal of Nursing Studies*, *48*(11), 1315–1322. https://doi.org/10.1016/j.ijnurstu.2011.04.005

Koniak-Griffin, D., Brecht, M.-L., Takayanagi, S., Villegas, J., Melendrez, M., & Balcázar, H. (2015). A community health worker-led lifestyle behavior intervention for Latina (Hispanic) women: feasibility and outcomes of a randomized controlled trial. *International Journal of Nursing Studies*, *52*(1), 75–87. https://doi.org/10.1016/j.ijnurstu.2014.09.005

Kontio, R., Lahti, M., Pitkänen, A., Joffe, G., Putkonen, H., Hätönen, H., … Välimäki, M. (2011). Impact of eLearning course on nurses’ professional competence in seclusion and restraint practices: a randomized controlled study (ISRCTN32869544). *Journal of Psychiatric and Mental Health Nursing*, *18*(9), 813–821. https://doi.org/10.1111/j.1365-2850.2011.01729.x

Kuo, S.-Y., Tsai, S.-H., Chen, S.-L., & Tzeng, Y.-L. (2016). Auricular acupressure relieves anxiety and fatigue, and reduces cortisol levels in post-caesarean section women: A single-blind, randomised controlled study. *International Journal of Nursing Studies*, *53*, 17–26. https://doi.org/10.1016/j.ijnurstu.2015.10.006

Kusahara, D. M., Peterlini, M. A. S., & Pedreira, M. L. G. (2012). Oral care with 0.12% chlorhexidine for the prevention of ventilator-associated pneumonia in critically ill children: randomised, controlled and double blind trial. *International Journal of Nursing Studies*, *49*(11), 1354–1363. https://doi.org/10.1016/j.ijnurstu.2012.06.005

Lai, H.-L., & Li, Y.-M. (2011). The effect of music on biochemical markers and self-perceived stress among first-line nurses: a randomized controlled crossover trial. *Journal of Advanced Nursing*, *67*(11), 2414–2424. https://doi.org/10.1111/j.1365-2648.2011.05670.x

Larsson, I., Fridlund, B., Arvidsson, B., Teleman, A., & Bergman, S. (2014). Randomized controlled trial of a nurse-led rheumatology clinic for monitoring biological therapy. *Journal of Advanced Nursing*, *70*(1), 164–175. https://doi.org/10.1111/jan.12183

Lee, H., Cumin, D., Devcich, D. A., & Boyd, M. (2015). Expressing concern and writing it down: an experimental study investigating transfer of information at nursing handover. *Journal of Advanced Nursing*, *71*(1), 160–168. https://doi.org/10.1111/jan.12484

Lee, K.-C., Chao, Y.-H., Yiin, J.-J., Chiang, P.-Y., & Chao, Y.-F. (2011). Effectiveness of different music-playing devices for reducing preoperative anxiety: a clinical control study. *International Journal of Nursing Studies*, *48*(10), 1180–1187. https://doi.org/10.1016/j.ijnurstu.2011.04.001

Lee, L.-C., Tsai, A. C., & Wang, J.-Y. (2015). Need-based nutritional intervention is effective in improving handgrip strength and Barthel Index scores of older people living in a nursing home: a randomized controlled trial. *International Journal of Nursing Studies*, *52*(5), 904–912. https://doi.org/10.1016/j.ijnurstu.2015.01.008

Lee, L.-C., Tsai, A. C., Wang, J.-Y., Hurng, B.-S., Hsu, H.-C., & Tsai, H.-J. (2013). Need-based intervention is an effective strategy for improving the nutritional status of older people living in a nursing home: a randomized controlled trial. *International Journal of Nursing Studies*, *50*(12), 1580–1588. https://doi.org/10.1016/j.ijnurstu.2013.04.004

Lee, M. K., Yun, Y. H., Park, H.-A., Lee, E. S., Jung, K. H., & Noh, D.-Y. (2014). A Web-based self-management exercise and diet intervention for breast cancer survivors: pilot randomized controlled trial. *International Journal of Nursing Studies*, *51*(12), 1557–1567. https://doi.org/10.1016/j.ijnurstu.2014.04.012

Lee, T.-Y., Chang, S.-C., Chu, H., Yang, C.-Y., Ou, K.-L., Chung, M.-H., & Chou, K.-R. (2013). The effects of assertiveness training in patients with schizophrenia: a randomized, single-blind, controlled study. *Journal of Advanced Nursing*, *69*(11), 2549–2559. https://doi.org/10.1111/jan.12142

Leung, S. S. K., & Lam, T. H. (2012). Group antenatal intervention to reduce perinatal stress and depressive symptoms related to intergenerational conflicts: a randomized controlled trial. *International Journal of Nursing Studies*, *49*(11), 1391–1402. https://doi.org/10.1016/j.ijnurstu.2012.06.014

Li, X.-M., Zhou, K.-N., Yan, H., Wang, D.-L., & Zhang, Y.-P. (2012). Effects of music therapy on anxiety of patients with breast cancer after radical mastectomy: a randomized clinical trial. *Journal of Advanced Nursing*, *68*(5), 1145–1155. https://doi.org/10.1111/j.1365-2648.2011.05824.x

Liao, W.-C., Wang, L., Kuo, C.-P., Lo, C., Chiu, M.-J., & Ting, H. (2013). Effect of a warm footbath before bedtime on body temperature and sleep in older adults with good and poor sleep: an experimental crossover trial. *International Journal of Nursing Studies*, *50*(12), 1607–1616. https://doi.org/10.1016/j.ijnurstu.2013.04.006

Liaw, J.-J., Yang, L., Katherine Wang, K.-W., Chen, C.-M., Chang, Y.-C., & Yin, T. (2012). Non-nutritive sucking and facilitated tucking relieve preterm infant pain during heel-stick procedures: a prospective, randomised controlled crossover trial. *International Journal of Nursing Studies*, *49*(3), 300–309. https://doi.org/10.1016/j.ijnurstu.2011.09.017

Liaw, J.-J., Yang, L., Lee, C.-M., Fan, H.-C., Chang, Y.-C., & Cheng, L.-P. (2013). Effects of combined use of non-nutritive sucking, oral sucrose, and facilitated tucking on infant behavioural states across heel-stick procedures: a prospective, randomised controlled trial. *International Journal of Nursing Studies*, *50*(7), 883–894. https://doi.org/10.1016/j.ijnurstu.2012.08.021

Lin, C.-H., Chiang, S.-L., Heitkemper, M. M., Hung, Y.-J., Lee, M.-S., Tzeng, W.-C., & Chiang, L.-C. (2016). Effects of telephone-based motivational interviewing in lifestyle modification program on reducing metabolic risks in middle-aged and older women with metabolic syndrome: A randomized controlled trial. *International Journal of Nursing Studies*, *60*, 12–23. https://doi.org/10.1016/j.ijnurstu.2016.03.003

Lourenço, L. B. de A., Rodrigues, R. C. M., Ciol, M. A., São-João, T. M., Cornélio, M. E., Dantas, R. A. S., & Gallani, M.-C. (2014). A randomized controlled trial of the effectiveness of planning strategies in the adherence to medication for coronary artery disease. *Journal of Advanced Nursing*, *70*(7), 1616–1628. https://doi.org/10.1111/jan.12323

Mak, S. S.-S., Lee, M.-Y., Cheung, J. S.-S., Choi, K.-C., Chung, T.-K., Wong, T.-W., … Lee, D. T. (2015). Pressurised irrigation versus swabbing method in cleansing wounds healed by secondary intention: a randomised controlled trial with cost-effectiveness analysis. *International Journal of Nursing Studies*, *52*(1), 88–101. https://doi.org/10.1016/j.ijnurstu.2014.08.005

McCann, T. V., Songprakun, W., & Stephenson, J. (2015). A randomized controlled trial of guided self-help for improving the experience of caring for carers of clients with depression. *Journal of Advanced Nursing*, *71*(7), 1600–1610. https://doi.org/10.1111/jan.12624

Moshki, M., Amiri, M., & Khosravan, S. (2012). Mental health promotion of Iranian university students: the effect of self-esteem and health locus of control. *Journal of Psychiatric and Mental Health Nursing*, *19*(8), 715–721. https://doi.org/10.1111/j.1365-2850.2011.01806.x

Moyle, W., Cooke, M. L., Beattie, E., Shum, D. H. K., O’Dwyer, S. T., & Barrett, S. (2014). Foot massage versus quiet presence on agitation and mood in people with dementia: a randomised controlled trial. *International Journal of Nursing Studies*, *51*(6), 856–864. https://doi.org/10.1016/j.ijnurstu.2013.10.019

Mujika, A., Forbes, A., Canga, N., de Irala, J., Serrano, I., Gascó, P., & Edwards, M. (2014). Motivational interviewing as a smoking cessation strategy with nurses: an exploratory randomised controlled trial. *International Journal of Nursing Studies*, *51*(8), 1074–1082. https://doi.org/10.1016/j.ijnurstu.2013.12.001

Navidian, A., & Bahari, F. (2014). The impact of mixed, hope and forgiveness-focused marital counselling on interpersonal cognitive distortions of couples filing for divorce. *Journal of Psychiatric and Mental Health Nursing*, *21*(7), 658–666. https://doi.org/10.1111/jpm.12058

Noben, C., Smit, F., Nieuwenhuijsen, K., Ketelaar, S., Gärtner, F., Boon, B., … Evers, S. (2014). Comparative cost-effectiveness of two interventions to promote work functioning by targeting mental health complaints among nurses: pragmatic cluster randomised trial. *International Journal of Nursing Studies*, *51*(10), 1321–1331. https://doi.org/10.1016/j.ijnurstu.2014.01.017

Ozdemir, S., Bebis, H., Ortabag, T., & Acikel, C. (2015). Evaluation of the efficacy of an exercise program for pregnant women with low back and pelvic pain: a prospective randomized controlled trial. *Journal of Advanced Nursing*, *71*(8), 1926–1939. https://doi.org/10.1111/jan.12659

Paquette, J., Le May, S., Lachance Fiola, J., Villeneuve, E., Lapointe, A., & Bourgault, P. (2013). A randomized clinical trial of a nurse telephone follow-up on paediatric tonsillectomy pain management and complications. *Journal of Advanced Nursing*, *69*(9), 2054–2065. https://doi.org/10.1111/jan.12072

Pu, Y., Cen, G., Sun, J., Gong, J., Zhang, Y., Zhang, M., … Fang, F. (2014). Warming with an underbody warming system reduces intraoperative hypothermia in patients undergoing laparoscopic gastrointestinal surgery: a randomized controlled study. *International Journal of Nursing Studies*, *51*(2), 181–189. https://doi.org/10.1016/j.ijnurstu.2013.05.013

Rambod, M., Sharif, F., Pourali-Mohammadi, N., Pasyar, N., & Rafii, F. (2014). Evaluation of the effect of Benson’s relaxation technique on pain and quality of life of haemodialysis patients: a randomized controlled trial. *International Journal of Nursing Studies*, *51*(7), 964–973. https://doi.org/10.1016/j.ijnurstu.2013.11.004

Rentala, S., Fong, T. C. T., Nattala, P., Chan, C. L. W., & Konduru, R. (2015). Effectiveness of body-mind-spirit intervention on well-being, functional impairment and quality of life among depressive patients - a randomized controlled trial. *Journal of Advanced Nursing*, *71*(9), 2153–2163. https://doi.org/10.1111/jan.12677

Saadatmand, V., Rejeh, N., Heravi-Karimooi, M., Tadrisi, S. D., Zayeri, F., Vaismoradi, M., & Jasper, M. (2013). Effect of nature-based sounds’ intervention on agitation, anxiety, and stress in patients under mechanical ventilator support: a randomised controlled trial. *International Journal of Nursing Studies*, *50*(7), 895–904. https://doi.org/10.1016/j.ijnurstu.2012.11.018

Sakellari, E., Sourander, A., Kalokerinou-Anagnostopoulou, A., & Leino-Kilpi, H. (2014). The impact of an educational mental health intervention on adolescents’ perceptions of mental illness. *Journal of Psychiatric and Mental Health Nursing*, *21*(7), 635–641. https://doi.org/10.1111/jpm.12151

Sawatzky, J.-A. V., Christie, S., & Singal, R. K. (2013). Exploring outcomes of a nurse practitioner-managed cardiac surgery follow-up intervention: a randomized trial. *Journal of Advanced Nursing*, *69*(9), 2076–2087. https://doi.org/10.1111/jan.12075

Schoonhoven, L., van Gaal, B. G. I., Teerenstra, S., Adang, E., van der Vleuten, C., & van Achterberg, T. (2015). Cost-consequence analysis of “washing without water” for nursing home residents: a cluster randomized trial. *International Journal of Nursing Studies*, *52*(1), 112–120. https://doi.org/10.1016/j.ijnurstu.2014.08.001

Seo, M., Kang, H. S., Lee, Y. J., & Chae, S. M. (2015). Narrative therapy with an emotional approach for people with depression: Improved symptom and cognitive-emotional outcomes. *Journal of Psychiatric and Mental Health Nursing*, *22*(6), 379–389. https://doi.org/10.1111/jpm.12200

Shao, J.-H., Chang, A. M., Edwards, H., Shyu, Y.-I. L., & Chen, S.-H. (2013). A randomized controlled trial of self-management programme improves health-related outcomes of older people with heart failure. *Journal of Advanced Nursing*, *69*(11), 2458–2469. https://doi.org/10.1111/jan.12121

Shorey, S., Chan, S. W. C., Chong, Y. S., & He, H.-G. (2015). A randomized controlled trial of the effectiveness of a postnatal psychoeducation programme on self-efficacy, social support and postnatal depression among primiparas. *Journal of Advanced Nursing*, *71*(6), 1260–1273. https://doi.org/10.1111/jan.12590

Shyu, Y.-I. L., Liang, J., Tseng, M.-Y., Li, H.-J., Wu, C.-C., Cheng, H.-S., … Yang, C.-T. (2013). Comprehensive and subacute care interventions improve health-related quality of life for older patients after surgery for hip fracture: a randomised controlled trial. *International Journal of Nursing Studies*, *50*(8), 1013–1024. https://doi.org/10.1016/j.ijnurstu.2012.11.020

Shyu, Y.-I. L., Liang, J., Tseng, M.-Y., Li, H.-J., Wu, C.-C., Cheng, H.-S., … Yang, C.-T. (2016). Enhanced interdisciplinary care improves self-care ability and decreases emergency department visits for older Taiwanese patients over 2 years after hip-fracture surgery: A randomised controlled trial. *International Journal of Nursing Studies*, *56*, 54–62. https://doi.org/10.1016/j.ijnurstu.2015.12.005

Songprakun, W., & McCann, T. V. (2012). Evaluation of a bibliotherapy manual for reducing psychological distress in people with depression: a randomized controlled trial. *Journal of Advanced Nursing*, *68*(12), 2674–2684. https://doi.org/10.1111/j.1365-2648.2012.05966.x

Songprakun, W., & McCann, T. V. (2012). Evaluation of a cognitive behavioural self-help manual for reducing depression: a randomized controlled trial. *Journal of Psychiatric and Mental Health Nursing*, *19*(7), 647–653. https://doi.org/10.1111/j.1365-2850.2011.01861.x

Spaniel, F., Novak, T., Bankovska Motlova, L., Capkova, J., Slovakova, A., Trancik, P., … Höschl, C. (2015). Psychiatrist’s adherence: a new factor in relapse prevention of schizophrenia. A randomized controlled study on relapse control through telemedicine system. *Journal of Psychiatric and Mental Health Nursing*, *22*(10), 811–820. https://doi.org/10.1111/jpm.12251

Stayt, L. C., Merriman, C., Ricketts, B., Morton, S., & Simpson, T. (2015). Recognizing and managing a deteriorating patient: a randomized controlled trial investigating the effectiveness of clinical simulation in improving clinical performance in undergraduate nursing students. *Journal of Advanced Nursing*, *71*(11), 2563–2574. https://doi.org/10.1111/jan.12722

Su, C.-P., Lai, H.-L., Chang, E.-T., Yiin, L.-M., Perng, S.-J., & Chen, P.-W. (2013). A randomized controlled trial of the effects of listening to non-commercial music on quality of nocturnal sleep and relaxation indices in patients in medical intensive care unit. *Journal of Advanced Nursing*, *69*(6), 1377–1389. https://doi.org/10.1111/j.1365-2648.2012.06130.x

Sun, G.-C., & Hsu, M.-C. (2016). Effects of nurse-led child- and parent-focused violence intervention on mentally ill adult patients and victimized parents: A randomized controlled trial. *International Journal of Nursing Studies*, *60*, 79–90. https://doi.org/10.1016/j.ijnurstu.2016.03.002

Taghizadeh, Z., Shirmohammadi, M., Feizi, A., & Arbabi, M. (2013). The effect of cognitive behavioural psycho-education on premenstrual syndrome and related symptoms. *Journal of Psychiatric and Mental Health Nursing*, *20*(8), 705–713. https://doi.org/10.1111/j.1365-2850.2012.01965.x

Tahir, N. M., & Al-Sadat, N. (2013). Does telephone lactation counselling improve breastfeeding practices? A randomised controlled trial. *International Journal of Nursing Studies*, *50*(1), 16–25. https://doi.org/10.1016/j.ijnurstu.2012.09.006

Tao, X., Chow, S. K. Y., & Wong, F. K. Y. (2015). A nurse-led case management program on home exercise training for hemodialysis patients: A randomized controlled trial. *International Journal of Nursing Studies*, *52*(6), 1029–1041. https://doi.org/10.1016/j.ijnurstu.2015.03.013

Teresi, J. A., Ramirez, M., Ellis, J., Silver, S., Boratgis, G., Kong, J., … Lachs, M. S. (2013). A staff intervention targeting resident-to-resident elder mistreatment (R-REM) in long-term care increased staff knowledge, recognition and reporting: results from a cluster randomized trial. *International Journal of Nursing Studies*, *50*(5), 644–656. https://doi.org/10.1016/j.ijnurstu.2012.10.010

Tiwari, A., Fong, D. Y. T., Wong, J. Y. H., Yuen, K., Yuk, H., Pang, P., … Bullock, L. (2012). Safety-promoting behaviors of community-dwelling abused Chinese women after an advocacy intervention: a randomized controlled trial. *International Journal of Nursing Studies*, *49*(6), 645–655. https://doi.org/10.1016/j.ijnurstu.2011.12.005

Tyrer, H., Tyrer, P., Lisseman-Stones, Y., McAllister, S., Cooper, S., Salkovskis, P., … Wang, D. (2015). Therapist differences in a randomised trial of the outcome of cognitive behaviour therapy for health anxiety in medical patients. *International Journal of Nursing Studies*, *52*(3), 686–694. https://doi.org/10.1016/j.ijnurstu.2014.11.013

Usher, K., Park, T., Foster, K., & Buettner, P. (2013). A randomized controlled trial undertaken to test a nurse-led weight management and exercise intervention designed for people with serious mental illness who take second generation antipsychotics. *Journal of Advanced Nursing*, *69*(7), 1539–1548. https://doi.org/10.1111/jan.12012

Utens, C. M. A., Goossens, L. M. A., van Schayck, O. C. P., Rutten-van Mölken, M. P. M. H., van Litsenburg, W., Janssen, A., … Smeenk, F. W. J. M. (2013). Patient preference and satisfaction in hospital-at-home and usual hospital care for COPD exacerbations: results of a randomised controlled trial. *International Journal of Nursing Studies*, *50*(11), 1537–1549. https://doi.org/10.1016/j.ijnurstu.2013.03.006

Utens, C. M. A., van Schayck, O. C. P., Goossens, L. M. A., Rutten-van Mölken, M. P. H. M., DeMunck, D. R. A. J., Seezink, W., … Smeenk, F. W. J. M. (2014). Informal caregiver strain, preference and satisfaction in hospital-at-home and usual hospital care for COPD exacerbations: results of a randomised controlled trial. *International Journal of Nursing Studies*, *51*(8), 1093–1102. https://doi.org/10.1016/j.ijnurstu.2014.01.002

van Gaal, B. G. I., Schoonhoven, L., Mintjes, J. A. J., Borm, G. F., Hulscher, M. E. J. L., Defloor, T., … van Achterberg, T. (2011). Fewer adverse events as a result of the SAFE or SORRY? programme in hospitals and nursing homes. part i: primary outcome of a cluster randomised trial. *International Journal of Nursing Studies*, *48*(9), 1040–1048. https://doi.org/10.1016/j.ijnurstu.2011.02.017

van Gaal, B. G. I., Schoonhoven, L., Mintjes, J. A. J., Borm, G. F., Koopmans, R. T. C. M., & van Achterberg, T. (2011). The SAFE or SORRY? programme. part II: effect on preventive care. *International Journal of Nursing Studies*, *48*(9), 1049–1057. https://doi.org/10.1016/j.ijnurstu.2011.02.018

Watson, H., Godfrey, C., McFadyen, A., McArthur, K., Stevenson, M., & Holloway, A. (2015). Screening and brief intervention delivery in the workplace to reduce alcohol-related harm: a pilot randomized controlled trial. *International Journal of Nursing Studies*, *52*(1), 39–48. https://doi.org/10.1016/j.ijnurstu.2014.06.013

Webster, J., Connolly, A., Paton, F., & Corry, J. (2011). The effectiveness of protocol drive, nurse-initiated discharge in a 23-h post surgical ward: a randomized controlled trial. *International Journal of Nursing Studies*, *48*(10), 1173–1179. https://doi.org/10.1016/j.ijnurstu.2011.02.022

Williams, A., Manias, E., Walker, R., & Gorelik, A. (2012). A multifactorial intervention to improve blood pressure control in co-existing diabetes and kidney disease: a feasibility randomized controlled trial. *Journal of Advanced Nursing*, *68*(11), 2515–2525. https://doi.org/10.1111/j.1365-2648.2012.05950.x

Wright, K., Giger, J. N., Norris, K., & Suro, Z. (2013). Impact of a nurse-directed, coordinated school health program to enhance physical activity behaviors and reduce body mass index among minority children: a parallel-group, randomized control trial. *International Journal of Nursing Studies*, *50*(6), 727–737. https://doi.org/10.1016/j.ijnurstu.2012.09.004

Wu, D. S., Hu, J., McCoy, T. P., & Efird, J. T. (2014). The effects of a breastfeeding self-efficacy intervention on short-term breastfeeding outcomes among primiparous mothers in Wuhan, China. *Journal of Advanced Nursing*, *70*(8), 1867–1879. https://doi.org/10.1111/jan.12349

Wu, L.-M., Chiou, S.-S., Sheen, J.-M., Lin, P.-C., Liao, Y. M., Chen, H.-M., & Hsiao, C.-C. (2014). Evaluating the acceptability and efficacy of a psycho-educational intervention for coping and symptom management by children with cancer: a randomized controlled study. *Journal of Advanced Nursing*, *70*(7), 1653–1662. https://doi.org/10.1111/jan.12328

Yan, J., You, L., Liu, B., Jin, S., Zhou, J., Lin, C., … Gu, J. (2014). The effect of a telephone follow-up intervention on illness perception and lifestyle after myocardial infarction in China: a randomized controlled trial. *International Journal of Nursing Studies*, *51*(6), 844–855. https://doi.org/10.1016/j.ijnurstu.2013.10.011

Yeh, H.-Y., Ma, W.-F., Huang, J.-L., Hsueh, K.-C., & Chiang, L.-C. (2016). Evaluating the effectiveness of a family empowerment program on family function and pulmonary function of children with asthma: A randomized control trial. *International Journal of Nursing Studies*, *60*, 133–144. https://doi.org/10.1016/j.ijnurstu.2016.04.013

Yin, T., Yang, L., Lee, T.-Y., Li, C.-C., Hua, Y.-M., & Liaw, J.-J. (2015). Development of atraumatic heel-stick procedures by combined treatment with non-nutritive sucking, oral sucrose, and facilitated tucking: a randomised, controlled trial. *International Journal of Nursing Studies*, *52*(8), 1288–1299. https://doi.org/10.1016/j.ijnurstu.2015.04.012

Zhang, M., Chan, S. W., You, L., Wen, Y., Peng, L., Liu, W., & Zheng, M. (2014). The effectiveness of a self-efficacy-enhancing intervention for Chinese patients with colorectal cancer: a randomized controlled trial with 6-month follow up. *International Journal of Nursing Studies*, *51*(8), 1083–1092. https://doi.org/10.1016/j.ijnurstu.2013.12.005

1. Zwijsen, S. A., Gerritsen, D. L., Eefsting, J. A., Smalbrugge, M., Hertogh, C. M. P. M., & Pot, A. M. (2015). Coming to grips with challenging behaviour: a cluster randomised controlled trial on the effects of a new care programme for challenging behaviour on burnout, job satisfaction and job demands of care staff on dementia special care units. *International Journal of Nursing Studies*, *52*(1), 68–74. https://doi.org/10.1016/j.ijnurstu.2014.10.003
